# Supplementary material for: Identification and Antimicrobial Resistance of Dermatophilus congolensis from Cattle in Saint Kitts and Nevis
Source: Vet Sci. 2021 Jul 16;8(7):135. doi: 10.3390/vetsci8070135 (PMC8310134; doi:10.3390/vetsci8070135)
Supplement: Supplementary file 1 [file vetsci-08-00135-s001.zip › vetsci-1259285-supplementary.pdf]

veterinary sciences

MDPI

**Table S1.** List of obtained isolates with MALDI-TOF species identification and score value.

| Sample ID | species ID | Score Value | Farm identifier |
|---|---|---|---|
| 19-027-761 Scab | *Dermatophilus congolensis* | 2.09 | St. Peters |
| 19-034-H1BF Swab | *Dermatophilus congolensis* | 1.75 | St. Peters |
| 19-034-H1BF Scab | *Dermatophilus congolensis* | 2.13 | St. Peters |
| 19-039-772 Scab | *Dermatophilus congolensis* | 2.09 | St. Pauls |
| 19-041-770 Scab | *Dermatophilus congolensis* | 1.99 | St. Pauls |
| 19-029-754 Scab | *Dermatophilus congolensis* | 2.01 | Old Road |
| 19-025-765 Scab | *Dermatophilus congolensis* | 1.77 | St. Peters |
| 19-023-762 Swab | *Dermatophilus congolensis* | 1.75 | St. Peters |
| 19-042-769 Scab | *Dermatophilus congolensis* | 2.00 | St. Pauls |
| 19-037-766 Scab | *Dermatophilus congolensis* | 1.89 | St. Pauls |
| 19-035-BF Swab | *Dermatophilus congolensis* | 1.85 | St. Peters |
| 19-032-762 Scab | *Dermatophilus congolensis* | 1.79 | St. Peters |
| 19-033-BF2 Swab | *Dermatophilus congolensis* | 2.07 | St. Peters |
| 19-040-358 Scab | *Dermatophilus congolensis* | 1.94 | St. Pauls |
| 19-015-76 Swab | *Dermatophilus congolensis* | 1.93 | Halfway Tree |
| 19-015-76 Scab | *Dermatophilus congolensis* | 1.72 | Halfway Tree |
| 19-018-Wilm Scab | *Dermatophilus congolensis* | 2.13 | Buckleys |
| 19-018-Wilm Swab | *Dermatophilus congolensis* | 1.89 | Buckleys |
| 19-021-Calv Swab | *Dermatophilus congolensis* | 2.17 | Buckleys |
| 19-021-Calv Scab | *Dermatophilus congolensis* | 1.85 | Buckleys |
| 19-020-Edri Scab | *Dermatophilus congolensis* | 1.82 | Buckleys |
| 19-023-762 Swab | *Dermatophilus congolensis* | 1.75 | St. Peters |
| 19-019-Muri Swab | *Dermatophilus congolensis* | 2.20 | Buckleys |
| 19-017-Clev Scab | *Dermatophilus congolensis* | 2.25 | Buckleys |
| 19-028-763 Swab | *Dermatophilus congolensis* | 1.81 | St. Peters |
| 19-026-764 Swab | *Dermatophilus congolensis* | 2.04 | St. Peters |
| 19-040-358 Swab | *Dermatophilus congolensis* | 2.11 | St. Pauls |
| 19-030-774 Swab | *Dermatophilus congolensis* | 1.81 | West Farm |
| 19-033-BF2 Scab | *Dermatophilus congolensis* | 2.01 | St. Peters |
| 19-030-774 Scab | *Dermatophilus congolensis* | 2.02 | West Farm |
| 19-035-BF Scab | *Dermatophilus congolensis* | 2.08 | St. Peters |
| 19-036-BF3 Scab | *Dermatophilus congolensis* | 1.75 | St. Peters |
| 19-032-H1BF2 Swab | *Dermatophilus congolensis* | 1.84 | St. Peters |
| 19-002-SBW Swab | *Dermatophilus congolensis* | 1.77 | Milliken |
| 19-002-SBW Scab | *Dermatophilus congolensis* | 1.73 | Milliken |
| 19-027-761 Swab | *Dermatophilus congolensis* | 1.87 | St. Peters |
| 19-024-767 Scab | *Dermatophilus congolensis* | 2.11 | St. Peters |
| 19-024-767 Swab | *Dermatophilus congolensis* | 1.82 | St. Peters |
| 19-028-763 Scab | *Dermatophilus congolensis* | 1.90 | St. Peters |
| 19-217-Clev Swab | *Dermatophilus congolensis* | 1.82 | Buckleys |
| 19-032-H1BF2 Swab | *Dermatophilus congolensis* | 1.98 | St. Peters |
| 19-019 Muri Scab | *Dermatophilus congolensis* | 2.17 | Buckleys |
| 19-003-MBF 1 Swab | *Dermatophilus congolensis* | 1.70 | Milliken |
